# The Association between Heme Oxygenase-1 Gene Promoter Polymorphism and the Outcomes of Catheter Ablation of Atrial Fibrillation

**DOI:** 10.1371/journal.pone.0056440

**Published:** 2013-02-20

**Authors:** Yu-Feng Hu, Kun-Tai Lee, Hsueh-Hsiao Wang, Kwo-Chang Ueng, Hung-I Yeh, Tze-Fan Chao, Jo-Nan Liao, Yenn-Jiang Lin, Shih-Lin Chang, Li-Wei Lo, Ta-Chuan Tuan, Cheng-Hung Li, Fa-Po Chung, Chiao-Po Hsu, Hsiao-Huang Chang, Cheng-Hsiung Huang, Shih-Ann Chen

**Affiliations:** 1 Division of Cardiology, Department of Medicine, Taipei Veterans General Hospital, Taipei, Taiwan; 2 Institute of Clinical Medicine and Cardiovascular Research Institute, National Yang-Ming University, Taipei, Taiwan; 3 Division of Cardiology, Department of Internal Medicine, Kaohsiung Medical University Hospital, Kaohsiung, Taiwan; 4 Departments of Internal Medicine and Medical Research, Mackay Memorial Hospital, Mackay Medical College, Taipei, Taiwan; 5 Chung Shan Medical University Hospital, Department of Medicine, School of Medicine, Chung Shan Medical University, Taichung, Taiwan; 6 Division of Cardiovascular Surgery, Department of Surgery, Taipei Veterans General Hospital, and National Yang-Ming University School of Medicine, Taipei, Taiwan; College of Pharmacy, University of Florida, United States of America

## Abstract

A length polymorphism of GT repeats in the promoter region of the human heme oxygenase-1 (HO-1) gene modulates its gene transcription to protect against myocardial injury. The present study investigated the association between HO-1 promoter polymorphisms and the outcomes of catheter ablation of atrial fibrillation (AF). The allelic frequencies of GT repeats in the HO-1 gene promoter were screened in 205 random individuals who underwent catheter ablation for drug refractory AF.In the patients who received catheter ablation, those with AF recurrence had fewer GT repeats (53.4±7.1 vs. 56.1±6.5, p = 0.004), a lower incidence of hyperlipidemia, more non-paroxysmal AF, and a larger left atrial diameter. After conducting a multivariate logistic analysis, the number of GT repeats (Odds ratio: 0.94, 95% CI 0.90–0.99, p = 0.01) and the diameter of the left atrium (Odds ratio: 1.08, 95% CI 1.02–1.15, p = 0.01) remained independent predictors. The carriers of GT repeats, which were <29 in both alleles, were associated with a lower sinus maintenance rate after catheter ablation (38.5% vs. 60.1%, p = 0.003). The patients were divided into paroxysmal and non-paroxysmal AF groups; the number of GT repeats was associated with AF recurrence only in the patients with paroxysmal AF. The number of GT repeats, combined with LAD, was significant for predicting AF recurrence after catheter ablation (p = 0.01). The number of GT repeats was not found to be associated with differences in the left atrial diameter, the biatrial voltage, or the levels of bilirubin, ferritin, iron, C-reactive protein, or von-Willibrand factor. In conclusions, HO-1 gene promoter polymorphisms were associated with AF recurrence after catheter ablation.

## Introduction

Previous studies have suggested that a length polymorphism of GT repeats in the promoter region of the human heme oxygenase-1 (HO-1) gene can modulate gene transcription and expression levels of HO-1. [1], [2], [3] As the GT repeats become shorter in the promoter region, HO-1 expression and activity increases. For example, reporter assays have demonstrated that the HO-1 promoter constructs that harbor (GT)_29_ or (GT)_38_ sequences were less active than those with (GT)_11_, (GT)_16_, (GT)_20,_ or (GT)_23_. [3], [4], [5], [6] In clinical studies, HO-1 promoter polymorphisms of GT repeats were associated with various cardiovascular diseases, such as coronary artery disease, restenosis after percutaneous transluminal angioplasty, and abdominal aortic aneurysms, in which different cut-points of GT repeats were used. [1], [7], [8], [9], [10].

The attempts by Husser et al. to relate genotypes to atrial fibrillation (AF) recurrence after pulmonary vein ablation procedures have shown a promising result, which suggested that genotypes could be a new tool to identify the patients at the risk of recurrence after catheter ablation. [11] HO-1 is an inducible isoform of the first and rate-controlling enzyme. This enzyme can degrade heme into iron, carbon monoxide, and biliverdin, which is subsequently converted into bilirubin. [1], [2], [12] HO-1 plays an important role in various stress-related pathophysiological states and affects anti-inflammatory, anti-oxidative, antiapoptotic, and cytoprotective functions, due to carbon monoxide and bilirubin. [1], [2], [12] These affected mechanisms are also involved in the recurrence of AF. In AF patients receiving catheter ablation, inflammatory markers, such as C-reactive protein and heat shock protein 27, and oxidative metabolites were associated with AF recurrence after catheter ablation. This association implied that oxidative stress and inflammation are potentially involved in the mechanisms of AF recurrence [13], [14], [15], [16], [17], [18], [19].

Therefore, we hypothesized that promoter polymorphism of HO-1 is associated with the outcomes after catheter ablation. The aim of the present study was to determine whether patients with AF recurrence after catheter ablation could have differences in their promoter allelic variants.

## Methods

### Ethics Statement

Ethical approval was obtained from the Institutional Review Board of the Veterans General Hospital, Taipei, Taiwan. All subjects gave written informed consent.

### Study Populations

This study involved 205 with drug-refractory AF received catheter ablation. The left atrial diameters (LAD) of the patients were determined before performing catheter ablation. Administration of all class I or III antiarrhythmic medications was discontinued at least five half-lives before the procedure. According to the 2006 American College of Cardiology/American Heart Association (ACC/AHA) guideline, recurrent AF is defined as paroxysmal if the arrhythmia terminates spontaneously. When sustained beyond 7 days, AF is defined as persistent. The category of persistent AF also includes cases of long-standing AF (e.g., greater than 1 year), usually leading to permanent AF, in which cardioversion has failed or has not been attempted. In the present study, persistent and permanent AF were classified as non-paroxysmal AF [20].

### Catheter Ablation Treatment of AF

The electrophysiological study and the contact electroanatomical mapping, catheter ablation of AF and the follow-up of recurrences were performed as described in our previous work. [13], [14], [15], [21] Administration of all class I or III antiarrhythmic medications was discontinued at least 5 half-lives before the procedure. In brief, each patient underwent an electrophysiological study and catheter ablation in the fasting, non-sedated state.

In the paroxysmal AF patients, we first tried to identify the spontaneous onset of the ectopy that triggered AF. The short-duration (8 beats) burst pacing from the right atrium, coronary sinus (CS), and pulmonary veins (PVs), with or without an isoproterenol infusion (up to 4 µg/min), was used to facilitate spontaneous AF. The PV ostia were identified by fluoroscopy and marked on a 3-dimensional map of the left atrium (LA). Continuous circumferential lesions encircling the right and left PV ostia were created by using 4-mm irrigated tip-ablation catheters guided by the NavX system (Therapy Cool Path, St Jude Medical, Inc., Minnetonka, MN). Radiofrequency ablation in the LA was performed at a power of 30 W on the anterior wall and a power of 25 W on the posterior wall. The tip of the catheter was irrigated with heparinized saline at the rate of 17 mL/min. After completion of the circumferential lesion set, the ipsilateral superior and inferior PVs were mapped carefully by using a circular recording catheter (Spiral, AF Division, St. Jude Medical, Inc., Minnetonka, MN) during sinus rhythm or CS pacing. After successful isolation of all 4 PVs, which was confirmed by PV circumferential mapping, high current (3–5 times the pacing threshold) and prolonged pulse stimulation (8 ms) of the proximal and distal CS were performed (in 10-ms decrements from 250–150 ms, with a 5–10 s pacing cycle length [CL] of 5–10 s) and repeated 3 to 5 times. In cases showing sustained AF/flutter, cardioversion was performed to restore sinus rhythm.

In non-paroxysmal AF patients, PV isolation was performed as the first step. If AF did not stop, then an additional ablation of the complex fractionated atrial electrogram (CFAE) sites was performed sequentially on the basis of the results of the CFAE maps obtained after pulmonary vein isolation (PVI). The endpoint of the ablation of the CFAE sites was prolongation of the CL, elimination of the CFAE sites, or abolishment of the local fractionated potentials (bipolar voltage, <0.05 mV). If the AF did not stop after additional ablation of the CFAE sites, then sinus rhythm was restored by performing electric cardioversion.

### Follow-up of AF Recurrences

After discharge, the patients underwent follow-up (2 weeks after the catheter ablation, then every 1–3 months thereafter at our cardiology clinic or with the referring physicians, where either 24-hour Holter monitoring or cardiac event recording with a recording duration of 1 week was performed, and antiarrhythmic drugs were prescribed for 8 weeks to prevent any early recurrence of AF. An AF recurrence was defined as an episode lasting more than 1 minute, and was confirmed by ECGs 3 months after the ablation (blanking period). The end point for the follow-up was the clinically documented recurrence of atrial arrhythmias or repeat ablation procedures.

### Genotyping of HO-1 Promoter

DNA was extracted from the buffy coat by using the high pure polymerase chain reaction (PCR) template preparation kit (Roche, Mannheim, Germany). The 5′-flanking region of the HO-1 gene containing a poly GT repeat was amplified by the polymerase chain reaction (PCR) using a fluorescent-labeled sense primer (5′-FAM-AGA GCC TGC AGC TTC TCA GA-3′), and an unlabeled antisense primer (5′-ACA AAG TCT GGC CAT AGG AC-3′). The sizes of PCR products were analyzed using an internal size-standard (GeneScan LIZ 500 size standard, Applied Biosystems, Foster City, CA), on a laser-based ABI 3730 DNA Analyzer (Applied Biosystems, Foster City, CA). Fragment length determination and GT-repeat length attribution was completed semi-automatically using ABI Prism Software (GeneMapper Version 3.7 Applied Biosystems, Foster City, CA).

### Statistical Analysis

Continuous variables are expressed as mean ± SD values. Comparisons between continuous variables were performed using the two-sample *t* test and Wilcoxon rank-sum test, as appropriate. Categorical data were compared using the Chi-square test. The logistic regression analysis was used to calculate odds ratios (ORs) and their 95% confidence intervals (CIs) in Table S1. Freedom from further AF was determined and compared using a Kaplan-Meier analysis and log-rank test (Figure S1). Cox multivariate regression and global chi-square test were used for Table S2 and Figure S2. A p-value less than 0.05 was considered statistically significant. This study used PSAW SPSS 18.0 for statistical analysis.

## Results

### The Number of GT Repeats was an Independent Predictor of AF Recurrence

A total of 205 patients were recruited from July 2008 to April 2011. The mean follow up duration was 582.6±272.2 days. The distribution of GT repeats in the present study was shown in Figure S1. As shown in Table S1, the patients with AF recurrence, compared with patients without AF recurrence, had a lower number of total GT repeats (the sum of GT repeats from 2 alleles, 53.4±7.1 vs. 56.1±6.5, p = 0.004), a lower incidence of hyperlipidemia (7.5% vs. 19.6%, p = 0.02), more non-paroxysmal AF (35.5% vs. 13.4%, p<0.001) and a larger LAD (41.2±7.7 vs. 38.3±5.0, p = 0.005). Age, gender distribution, incidence of diabetes mellitus, hypertension, coronary artery disease, heart failure history, statins, and angiotensin converting enzyme inhibitor/angiotensin receptor blocker did not differ between the AF and control groups. After conducting a multivariate logistic analysis, the number of GT repeats (Odds ratio: 0.94 per GT repeat, 95% CI 0.90–0.99, p = 0.01) and LAD (Odds ratio: 1.08, 95% CI 1.02–1.15, p = 0.01) remained independently associated with AF recurrence when adjusted for age, sex, hyperlipidemia, statins, angiotensin converting enzyme inhibitor/angiotensin receptor blocker, and the incidence of non-paroxysmal AF.

According to a previous report, GT repeats <29 were more active in the function and expression of HO-1 than GT repeats ≥29, and the sensitivity and specificity test were provided in the Table S1. [3] Similarly, the GT repeat number of 29 was suggested as the best cut point by Youden’s index. Therefore, we further divided the patients into two groups (GT repeats<29 in both alleles [n = 52], and GT repeats≥29 in any allele[n = 153]). In all AF patients, the carriers of GT repeats <29 in both alleles were associated with a lower sinus maintenance rate after catheter ablation (38.5% vs. 60.1%; mean survival time: 612 days, as seen in Figure S2).

After conducting a Cox multivariate regression, the carriers of GT repeats <29 in both alleles were still independently associated with a higher AF recurrence after catheter ablation (Table S2) in all patients. We further divided the patients into paroxysmal and non-paroxysmal AF groups. We observed that the number of GT repeats was associated with AF recurrence only in patients with paroxysmal AF. The p-interaction between GT number and paroxysmal AF vs. non-paroxysmal AF was 0.33.

Combining the assessment of HO-1 GT repeats and LAD could help to characterize the risk of AF recurring after catheter ablation (Figure S3). In patients with concomitant LAD ≥40 mm and GT repeats <29 in both alleles, the AF recurrence rate was 82.6%. In contrast, the patients who had an LAD <40 mm and GT repeats ≥29 in any allele had the lowest AF recurrence rate (32.5%). The addition of GT repeats to a Cox model with LAD resulted in a significant improvement of the global Chi-square value (13.84 vs. 23.27, p = 0.01), which illustrated the incremental value of HO-1 repeats, combined with LAD, for correlating AF recurrence after catheter ablation. Adjusted hazard ratio by the combination of HO-1 repeats and LAD was higher than those by LAD alone (2.93 vs. 1.40).

### The Associated Mechanisms of the Number of GT Repeats

We correlated the number of GT repeats to possible mediators to identify possible mechanisms (Table S3). Compared to the carriers of GT repeats ≥29 in any allele, those with GT repeats <29 in both alleles were not associated with different LAD, left atrial, or right atrial voltage. The bilirubin-andiron-associated pathways were suggested as one of the mediators; however, the carriers of GT repeats <29 in both alleles were not associated with different levels of total and direct bilirubin, iron, and ferritin, as compared with the carriers of GT repeats ≥29 in any allele. Furthermore, levels of nitrate/nitrite, HsCRP, and VWF did not differ.

## Discussion

### Major Findings

To our best knowledge, this is the first report stating that GT repeats of HO-1 gene are associated with AF recurrence after catheter ablation. Lower GT repeats, which indicate higher HO-1 activity and expression, were associated with a higher AF recurrence after catheter ablation and suggest that HO-1 offers a protective effect against ablation-induced myocardial damage. This mechanism is not associated with bilirubin or any iron-associated pathway.

### HO-1 Promoter Polymorphisms and AF Recurrence after Catheter Ablation

Heme oxygenase-1 is a protective factor potential anti-inflammatory, anti-oxidative, and anti-apoptosis effects. [2], [12], [22] In animal models, HO-1 in transgenic mice prevented angiotensin II-induced high reactive oxygen species and inflammatory cytokines in vascular smooth muscle, adjacent endothelial, and cardiomyocytes. [23] HO-1 over-expression by adenovirus-mediated transfection into rat hearts increased the anti-apoptotic Bcl-2 protein, decreased lipid peroxidation, proapoptotic Bax, and proinflammatory interleukin-1-beta protein levels in a myocardial infarction model [24].

Promoter polymorphisms (GT repeats) of HO-1 are associated with transcription of the HO-1 gene, and short GT repeats lead to high HO-1 expression and enzyme activity. [1], [2], [3], [12] In clinical studies, patients with short GT repeats exhibited a significantly reduced level of inflammation following balloon angioplasty, compared to carriers of long GT repeats. [25] Leukocyte HO-1 gene expression has also been found to negatively correlate to markers of oxidative stress in patients with diabetic microangiopathy [26].

Catheter ablation leads to permanent hyperthermia-associated damage of cardiac tissue and creates a conduction block between the pulmonary vein and the atrium. Specifically, the cardiomyocytes along the isolation line must be destroyed for the procedure to be successful. [20] Hyperthermia causes a rapid increase of HO-1 expression, heme degradation capacity, and tissue and cyclic GMP levels in the atrium, when measured one hour post-treatment [27].

HO-1 plays an important cardio-protective role in adapting to various stresses, such as hyperthermia, and exerting potential anti-inflammatory, antioxidative, and anti-apoptosis effects. [1], [12], [22] For example, in different animal models, such as coronary ligation-induced heart failure or ischemic/reperfusion-induced cardiac dysfunction, HO-1 induction prevents oxidative stress, fibrosis, and apoptosis in the stressed heart. [28], [29] Through these protective mechanisms, it is possible that HO-1 plays an auto-defensive role during hyperthermia, preventing ablation-induced hyperthermia-related damage in the cardiomyocytes. Subsequently, this protection leads to a higher pulmonary vein reconnection and AF recurrence in patients with paroxysmal AF. However, future studies are necessary to confirm this hypothesis. The HO-1 gene could be a therapeutic target through gene transference or pharmacological modulation to prevent recurrent AF after catheter ablation [22], [24].

The ablation strategies were different in the patients with non-paroxysmal AF and paroxysmal AF. In addition to pulmonary vein isolation, extensive ablation applying to area with complex fractionated atrial electrograms was performed in the patients with non-paroxysmal AF. Furthermore, non-paroxysmal AF was associated with more atrial remodeling, worse atrial voltage, and more non-pulmonary trigger, compared to paroxysmal AF. The sinus maintenance rate after a single procedure in paroxysmal AF is around 70%, and those for non-paroxysmal AF could be as low as 20%. [20], [30], [31] Therefore, subgroup analysis (paroxysmal and non-paroxysmal AF) was performed. The HO-1 promoter polymorphism was not associated with different outcome after catheter ablation in the patients with non-paroxysmal AF. However, we need to be cautious when interpreting this result because the power for statistical analysis in the patients with non-paroxysmal AF might be insufficient due to the limited case numbers.

### Possible Mediators of HO-1 Promoter Polymorphisms

HO-1 degrades heme into iron, carbon monoxide, and biliverdin, which is subsequently converted into bilirubin. [1], [2], [12], [22] Furthermore, HO-1 can increase ferritin levels via different pathways. [32] Several positive biological effects, such as anti-inflammatory, antiapoptotic, and anti-oxidative effects, are partially attributable to carbon monoxide, bilirubin, and possibly, ferritin. [1], [2], [12], [22], [32] Length polymorphism in the HO-1 gene promoter is correlated with a risk of coronary artery disease in diabetic patients. This effect may be explained by its influence on serum bilirubin and ferritin. [7] Promoter polymorphism has also been correlated with an increase of CRP after balloon angioplasty in the coronary arteries. [25] However, in the present study, GT repeats were not associated with levels of bilirubin, ferritin, and iron. Furthermore, GT repeats were not associated with inflammatory markers (hsCRP and VWF) or markers for endothelial function (nitrite/nitrate). These findings suggest that the role of HO-1 in the pathogenesis of AF may not be the same as its role in coronary artery disease. However, insignificant correlations between serum markers did not exclude the possibility of interactions at the cellular level. The present study did not evaluate the level of carbon monoxide, which decays rapidly within seconds; therefore, it is difficult to obtain a reliable concentration measurement.

### Limitations

The distribution of GT repeats in the present study was similar to those studies from Japan and Austria, and the GT repeat numbers of 30 or 23 are the most predominant genotypes regardless of the ethnic background. [9], [33] The findings in the present study might be applied to other ethnic populations based on similar distribution of HO-1 promoter polymorphism. However, a large-scale study may be required to replicate the relationship between HO-1 promoter polymorphisms and AF recurrence in an independent population. Furthermore, the ablation strategies in different centers might be different; which could prevent the generalization of the present study. The precise mechanisms for this relationship are not clear, and future studies may be needed to delineate the possible signal pathways observed in AF pathogenesis. The 12-lead ECG at each follow-up visit was not regularly performed in the beginning of the present study, which is not in concordance with the present guideline.

### Conclusions

HO-1 gene promoter polymorphisms were associated with AF and its recurrence after catheter ablation.

## Supporting Information

Figure S1 **The distribution of the number of GT repeats.** The incidences of GT number of 29 and 23 were higher than the others.
(TIF)

Figure S2 **The number of GT repeats and AF recurrence after catheter ablation.** GT repeats <29 in both alleles were associated with a lower sinus rhythm maintenance rate after catheter ablation.
(TIF)

Figure S3 **The outcomes of catheter ablation according to GT repeats and LAD.** HO-1 GT repeats <29 in both alleles, combined with LAD, were significant in predicting AF recurrence after catheter ablation.
(TIF)

Table S1 **The predictors of AF recurrence after catheter ablation.**
(DOCX)

Table S2 **The association of HO-1 promoter genotypes (GT number <29 in both alleles) and atrial fibrillation recurrence after catheter ablation.**
(DOCX)

Table S3 **The relationships between seral markers and HO-1 GT repeat numbers.**
(DOCX)
