# Supplementary figures and images for: The Association between Heme Oxygenase-1 Gene Promoter Polymorphism and the Outcomes of Catheter Ablation of Atrial Fibrillation
Source: PLoS One. 2013 Feb 20;8(2):e56440. doi: 10.1371/journal.pone.0056440 (PMC3577889; doi:10.1371/journal.pone.0056440)

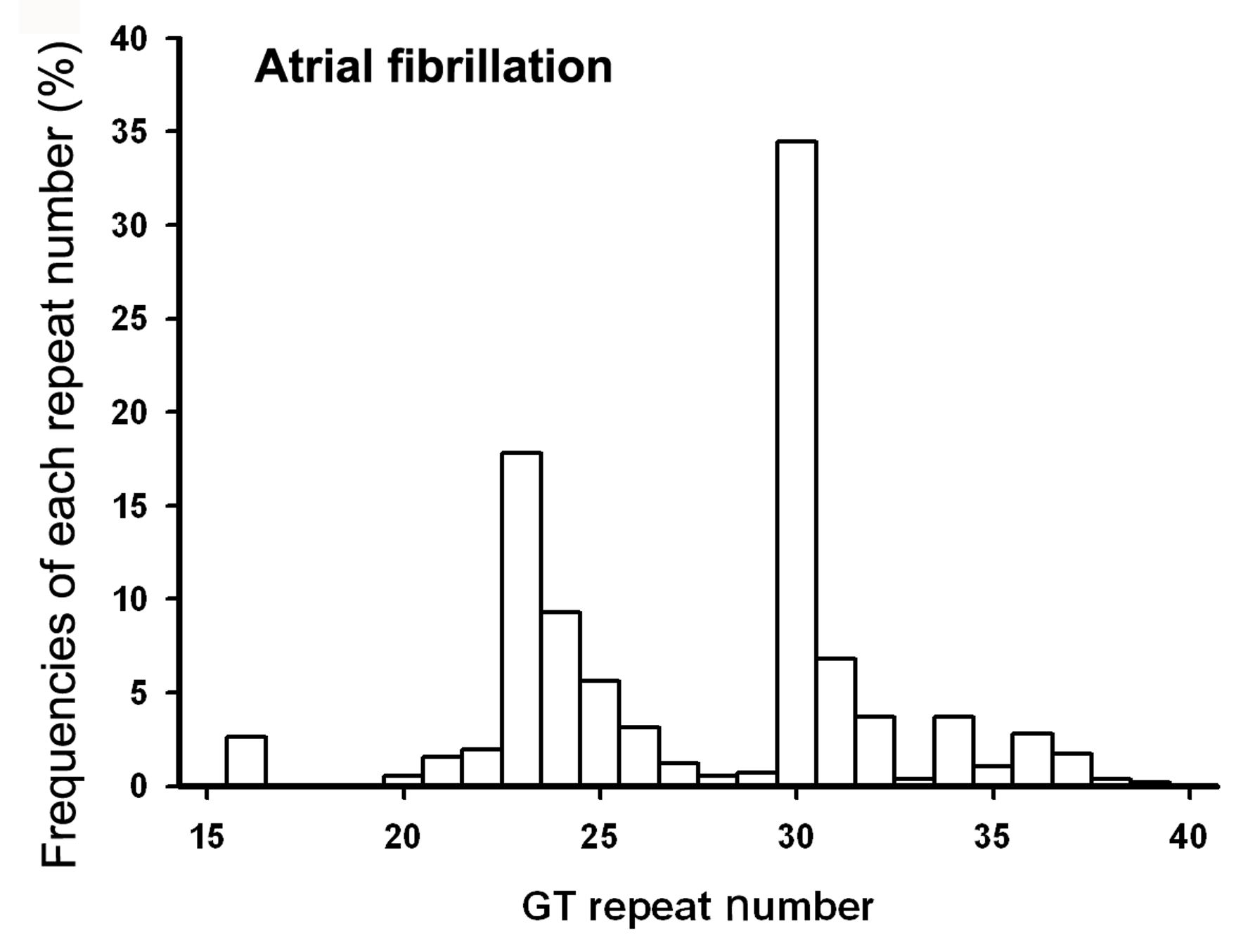

Supplement: Figure S1 — The distribution of the number of GT repeats. The incidences of GT number of 29 and 23 were higher than the others. (TIF) [file pone.0056440.s001.tif]

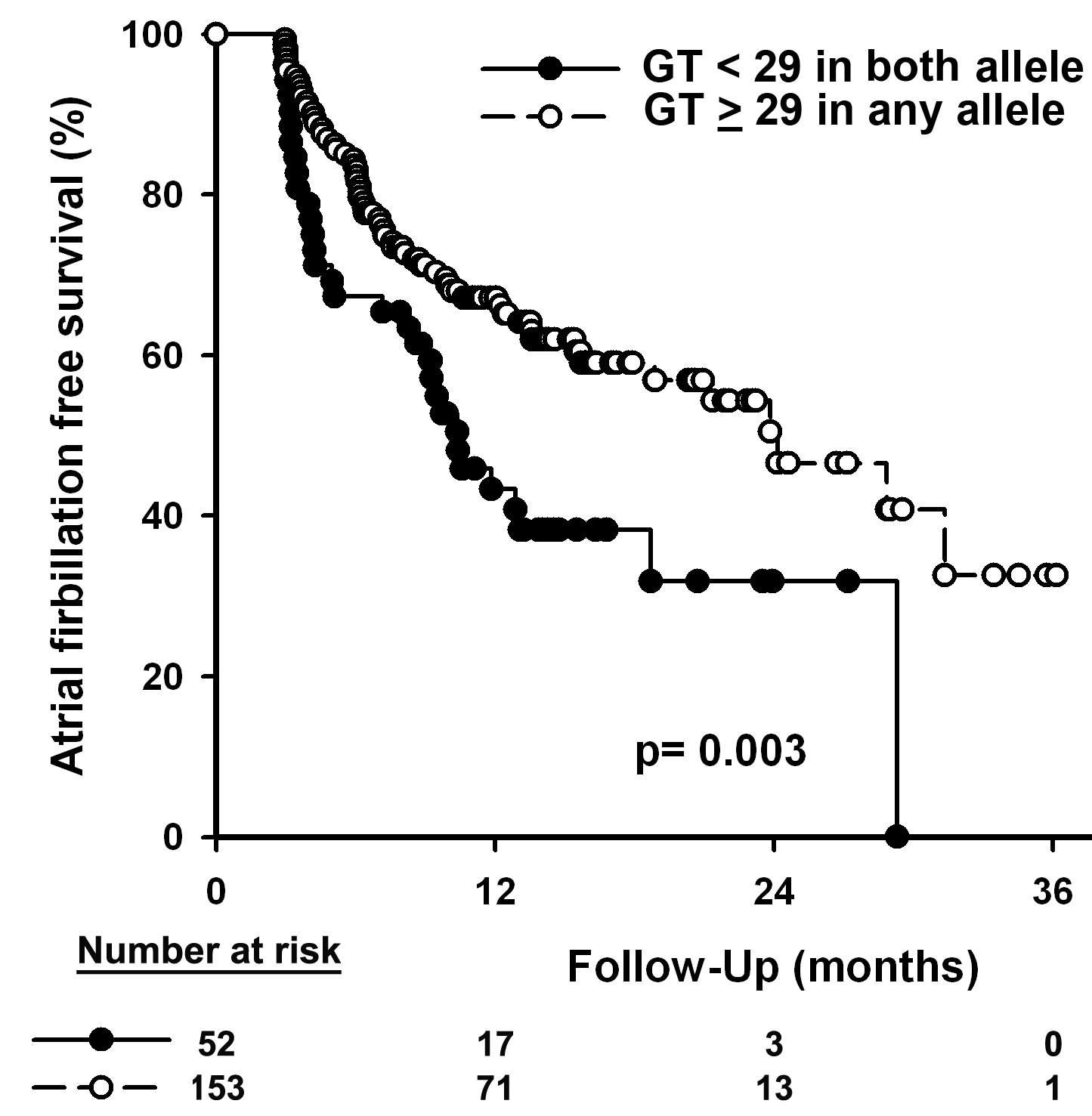

Supplement: Figure S2 — The number of GT repeats and AF recurrence after catheter ablation. GT repeats <29 in both alleles were associated with a lower sinus rhythm maintenance rate after catheter ablation. (TIF) [file pone.0056440.s002.tif]

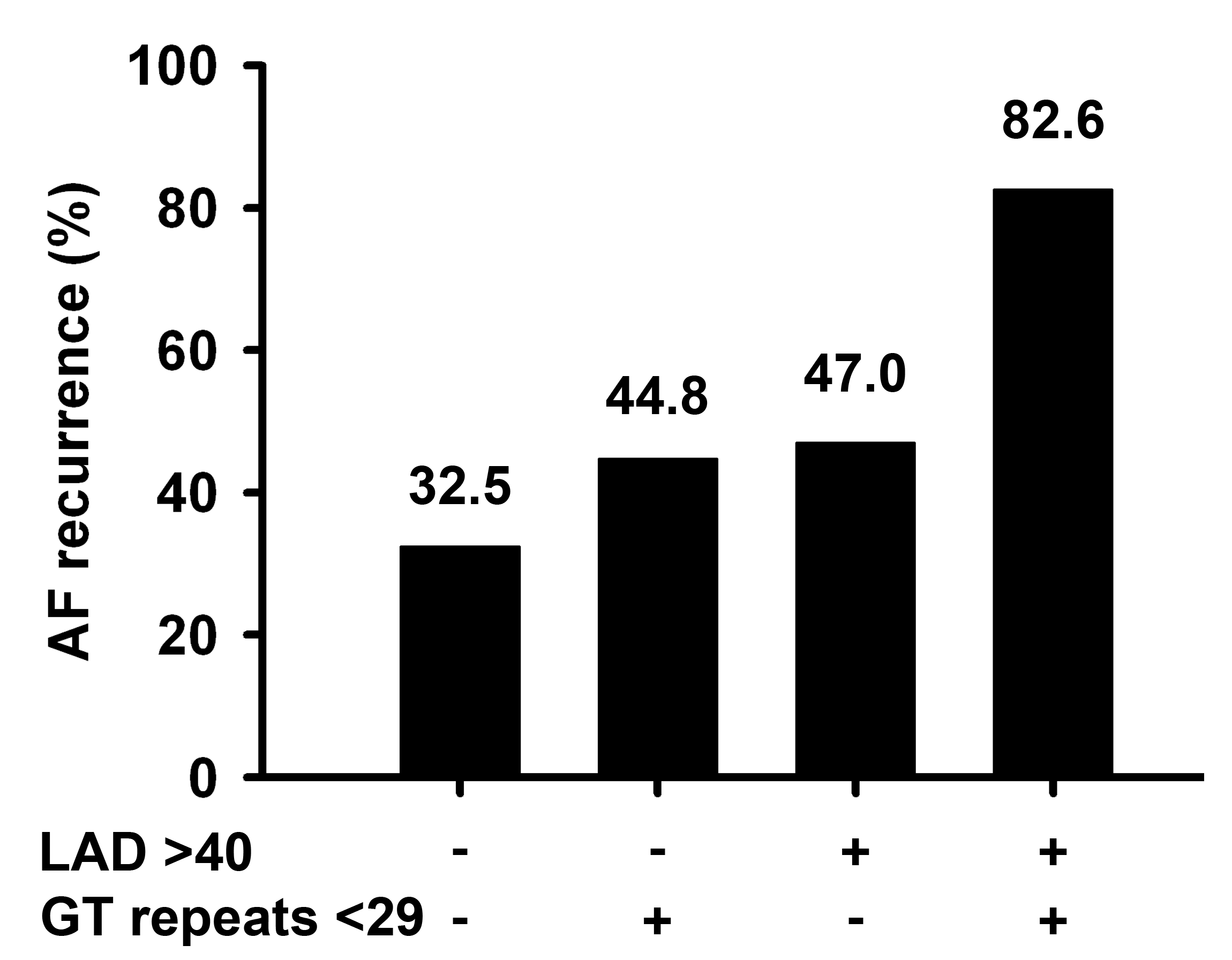

Supplement: Figure S3 — The outcomes of catheter ablation according to GT repeats and LAD. HO-1 GT repeats <29 in both alleles, combined with LAD, were significant in predicting AF recurrence after catheter ablation. (TIF) [file pone.0056440.s003.tif]
