# Supplementary material for: The Association between Heme Oxygenase-1 Gene Promoter Polymorphism and the Outcomes of Catheter Ablation of Atrial Fibrillation
Source: PLoS One. 2013 Feb 20;8(2):e56440. doi: 10.1371/journal.pone.0056440 (PMC3577889; doi:10.1371/journal.pone.0056440)
Supplement: Table S1 — The predictors of AF recurrence after catheter ablation. (DOCX) [file pone.0056440.s004.docx]

**Table S1. The predictors of AF recurrence after catheter ablation**

|  | Recurrence | No recurrence | Univariate | Multivariate | |
| --- | --- | --- | --- | --- | --- |
|  | N=93 | N=112 | p-value | Odds ratio (95% CI) | p-value |
| Age (year) | 52.7±10.3 | 55.1±10.3 | 0.10 |  | 0.14 |
| Sex (male) | 73 (78.5%) | 74(66.1%) | 0.07 |  | 0.84 |
| Diabetes Mellitus | 5(5.4%) | 10(8.9%) | 0.48 |  |  |
| Hypertension | 29(31.2%) | 46(41.1%) | 0.19 |  |  |
| Coronary artery disease | 5(5.4%) | 6(5.4%) | 0.99 |  |  |
| Heart failure history | 2(2.2%) | 0(0%) | 0.40 |  |  |
| Hyperlipidemia | 7 (7.5%) | 22(19.6%) | 0.02 |  | 0.12 |
| Statins | 6 (6.5%) | 17(15.2%) | 0.08 |  | 0.41 |
| ACEI/ARB | 16(17.2%) | 23(20.5%) | 0.67 |  | 0.48 |
| Left atrial diameter (mm) | 41.2±7.7 | 38.3±5.0 | 0.005 | 1.08 (1.02-1.15) | 0.01 |
| Non-paroxysmal AF | 33(35.5%) | 15(13.4%) | <0.001 |  | 0.37 |
| The number of GT repeats | 53.4±7.1 | 56.1±6.5 | 0.004 | 0.94 (0.90-0.99) | 0.01 |

ACEI/ARB: angiotensin converting enzyme inhibitor/angiotesin receptor blocker; HO-1: heme oxygenase-1
