# Supplementary material for: The Association between Heme Oxygenase-1 Gene Promoter Polymorphism and the Outcomes of Catheter Ablation of Atrial Fibrillation
Source: PLoS One. 2013 Feb 20;8(2):e56440. doi: 10.1371/journal.pone.0056440 (PMC3577889; doi:10.1371/journal.pone.0056440)
Supplement: Table S2 — The association of HO-1 promoter genotypes (GT number <29 in both alleles) and atrial fibrillation recurrence after catheter ablation. (DOCX) [file pone.0056440.s005.docx]

**Table S2. The association of HO-1 promoter genotypes (GT number <29 in both alleles) and atrial fibrillation recurrence after catheter ablation**

|  | Hazard ratio (95% CI) | p-value |
| --- | --- | --- |
| **Total AF (n=205)** |  |  |
| Non-adjusted model | 1.89 (1.23-2.94) | 0.004 |
| Adjusted by age, sex and underlying diseases | 1.72 (1.11-2.70) | 0.01 |
| Additionally adjusted for LAD | 1.79 (1.10-2.94) | 0.02 |
| **Paroxysmal AF (n=157)** |  |  |
| Non-adjusted model | 2.00 (1.15-3.45) | 0.01 |
| Adjusted by age, sex and underlying diseases | 1.82 (1.04-3.23) | 0.03 |
| Additionally adjusted for LAD | 1.92 (1.05-4.39) | 0.03 |
| **Non-paroxysmal AF (n=48)** |  |  |
| Non-adjusted model | 1.25 (0.63-2.50) | 0.52 |

**Underlying diseases:** Diabetes Mellitus, hypertension, coronary artery disease, heart failure history, and hyperlipidemia; LAD: left atrial diameter
