# Supplementary material for: The Association between Heme Oxygenase-1 Gene Promoter Polymorphism and the Outcomes of Catheter Ablation of Atrial Fibrillation
Source: PLoS One. 2013 Feb 20;8(2):e56440. doi: 10.1371/journal.pone.0056440 (PMC3577889; doi:10.1371/journal.pone.0056440)
Supplement: Table S3 — The relationships between seral markers and HO-1 GT repeat numbers. (DOCX) [file pone.0056440.s006.docx]

**Table S3. The relationships between seral markers and HO-1 GT repeat numbers**

|  | GT repeats ≥ 29  in any allele (N=153) | GT repeats < 29  in both allele (N=52) | p value |
| --- | --- | --- | --- |
| LAD (mm) | 39.2±6.2 | 40.5±7.5 | 0.25 |
| LA voltage (mV) | 2.10±0.68 | 1.93±0.60 | 0.19 |
| RA voltage (mV) | 2.02±0.57 | 1.97±0.56 | 0.63 |
| Total bilirubin (mg/dL) | 0.79±0.33 | 0.76±0.28 | 0.68 |
| Direct bilirubin (mg/dL) | 0.21±0.12 | 0.22±0.09 | 0.67 |
| Iron (μg/dL) | 86.13±31.02 | 87.53±32.03 | 0.80 |
| Ferritin (μg/dl) | 202.13±166.47 | 190.66±122.92 | 0.68 |
| Nitrate/nitrite (uM) | 58.90±33.35 | 60.35±39.06 | 0.85 |
| HsCRP (mg/dl) | 0.14±0.19 | 0.22±0.44 | 0.23 |
| VWF (mu/ml) | 10.25±4.77 | 11.11±4.38 | 0.28 |

RA: right atrium, LA: left atrium, LAD: left atrial diameter, HsCRP: high sensitive CRP
